# The status of *Her2* amplification and *Kras* mutations in mucinous ovarian carcinoma

**DOI:** 10.1186/s40246-016-0096-9

**Published:** 2016-12-28

**Authors:** Kuang-Leei Chang, Ming-Yung Lee, Wan-Ru Chao, Chih-Ping Han

**Affiliations:** 1 Department of Emergency Medicine, Chung-Shan Medical University and Chung Shan Medical University Hospital, Taichung, Taiwan; 2 Department of Statistics and Informatics Science, Providence University, Taichung, Taiwan; 3 Department of Pathology, Chung-Shan Medical University and Chung Shan Medical University Hospital, Taichung, Taiwan; 4 Department of Obstetrics and Gynecology, Chung-Shan Medical University and Chung-Shan Medical University Hospital, No 110, Sec 1, Chien Kuo N. Rd, Taichung, 40201 Taiwan

**Keywords:** *Kras* mutation, *Her2* amplification, Mucinous ovarian carcinoma

## Abstract

Jayson GC et al. remarked in *Lancet* that nearly 100% of mucinous ovarian cancer cases have *Kras* mutation as well as a high frequency of *Her2* amplification. Using the Abbott PathVysion *Her2* DNA Probe Kit and *Kras* mutant-enriched PCR Kits (FemtoPath®), 21 samples of primary ovarian mucinous cystadenocarcinomas from Taiwanese patients were examined to determine the status of *Her2* amplification and *Kras* mutations. Our results showed the *Her2* amplification rates were 33.33%, while the *Kras* mutation rates were 61.90%. We present here our results in order to enlighten the readership that the ~100% *Kras* mutant frequency and the high *Her2* amplification rate reported by Jayson et al. may be too exaggerated to be applicable into all populations. Additionally, we report another 2 novel *Kras* mutations (A11V, V14I).

## Main text

We read with great interest the work by Jayson et al. in *Lancet* (Oct. 2014). The authors presented a comprehensive review of outstanding quality. They remarked that mucinous ovarian carcinoma has a nearly 100% human V-Ki-ras2 Kirsten rat sarcoma viral oncogene homolog (*Kras*) mutation as well as a high frequency of human epidermal growth factor receptor 2 (*Her2*) amplification [1]. However, we respectfully disagree with Jayson et al.’s opinion.

Literature reviews revealed that in mucinous ovarian carcinoma, the frequency of *Her2* amplification/over-expression is estimated to be between 18 and 35% [2], and the presence of human *Kras* mutations is 13 to 60% [3–5]. This preliminary report aims to enlighten the readership that the ~100% *Kras* mutant frequency and the high *Her2* amplification rate in mucinous ovarian carcinoma may be higher than what has been observed in other studies, including our own.

Briefly, genomic DNA was extracted from formalin-fixed, paraffin-embedded tissue blocks of 21 cases of mucinous ovarian carcinoma. All the donors’ identities have been permanently deleted. Abbott PathVysion *Her2* DNA Probe Kit and the 2013 American Society of Clinical Oncology/College of American Pathologists (ASCO/CAP) breast cancer scoring methods were used to examine for *Her2* FISH ratio. The *Kras* mutant-enriched polymerase chain reaction (PCR) Kits (FemtoPath®) and a following direct sequencing method were applied to analyze exon 2 of the *Kras* gene. The reason why we choose *Kras* exon 2 to analyze is because *Kras* gene mutations are mainly known to cluster in several hotspots, with exon 2 (codons 12 and 13) being most commonly affected [6–9].

The prevalence of *Kras* mutations and *Her2* amplification within 21 Taiwanese mucinous ovarian carcinoma cases is shown in Table 1, which indicates that the amplification rate of *Her2* was 33.33% (*n* = 7), while the mutation rate of *Kras* was 61.90% (*n* = 13). Additionally, the rates of co-existing *Kras* mutations and *Her2* amplification were 9.52% (*n* = 2) (Table 1). However, there was a lack of statistically significant association between *Her2* amplification and *Kras* mutations (*p* = 0.057).

**Table 1:** The prevalence and relationship of *Kras* mutations and *Her2* amplification in mucinous ovarian carcinoma

	*Her2* non-amplification	*Her2* amplification	Total
	*n* (%)	*n* (%)	*n* (%)
*Kras* wild type	3 (14.29)	5 (23.81)	8 (38.10)
*Kras* mutation	11 (52.38)	2 (9.52)	13 (61.90)
Total	14 (66.67)	7 (33.33)	21 (100.00)
*P* value	0.056^a^		

*n (%)* number (percentage)

^a^Fisher’s exact test

Of the 13 cases of mucinous ovarian carcinoma with *Kras* mutations, 12 cases had a single missense mutation, which was composed of G12V in 4 cases, G12D in 5 cases, G12A in 1 case and A11V in 2 cases. The remaining 1 case had triple missense mutations—A11V, G13N and V14I. Moreover, both A11V and V14I were novel discoveries, based on the Catalogue of Somatic Mutations in Cancer (COSMIC) database [10].

## Conclusion

Both *Her2* amplification and *Kras* activating mutations are not mutually exclusive, which indicates that *Her2*/*Kras*/mitogen-activated protein kinases (MAPK) is a crucial pathway in the carcinogenesis of mucinous ovarian neoplasms. Targeting this pathway seems to be a viable therapeutic option for patients with recurrent or advanced stage mucinous ovarian carcinoma. Treatment selection based on the molecular alterations of *Her2* and *Kras* can possibly produce superior therapeutic effects compared with nonselective treatments. Additionally, functional impacts of these 2 novel *Kras* mutations (A11V, V14I) are still unknown; further studies using bioinformatics tools and molecular modeling are encouraged.

## Abbreviations

ASCO/CAP: American Society of Clinical Oncology/College of American Pathologists
COSMIC: Catalogue of Somatic Mutations in Cancer
*Her2*: Human epidermal growth factor receptor 2 gene
ICH: International Conference on Harmonization
*Kras*: V-Ki-ras2 Kirsten rat sarcoma viral oncogene homolog gene
MAPK: Mitogen-activated protein kinases
PCR: Polymerase chain reaction
